# Janus-Faced Neutrophil Extracellular Traps in Periodontitis

**DOI:** 10.3389/fimmu.2017.01404

**Published:** 2017-10-26

**Authors:** Ljubomir Vitkov, Dominik Hartl, Bernd Minnich, Matthias Hannig

**Affiliations:** ^1^Department of Cell Biology and Physiology, Division of Animal Structure and Function, University of Salzburg, Salzburg, Austria; ^2^Clinic of Operative Dentistry, Periodontology and Preventive Dentistry, Saarland University, Homburg, Germany; ^3^Department of Paediatrics, Paediatric Infectiology, Immunology and Cystic Fibrosis, Children’s Hospital, University of Tübingen, Tübingen, Germany

**Keywords:** neutrophils, lipopolysaccharide, Papillon–Lefèvre syndrome, chronic granulomatous disease, bystander damages, NETosis

## Abstract

Periodontitis is characterized by PMN infiltration and formation of neutrophil extracellular traps (NETs). However, their functional role for periodontal health remains complex and partially understood. The main function of NETs appears to be evacuation of dental plaque pathogen-associated molecular patterns. The inability to produce NETs is concomitant with aggressive periodontitis. But in cases with exaggerated NET production, NETs are unable to maintain periodontal health and bystander damages occur. This pathology can be also demonstrated in animal models using lipopolysaccharide as PMN activator. The progress of periodontitis appears to be a consequence of the formation of gingival pockets obstructing the evacuation of both pathogen-associated and damage-associated molecular patterns, which are responsible for the self-perpetuation of inflammation. Thus, besides the pathogenic effects of the periodontal bacteria, the dysregulation of PMN activation appears to play a main role in the periodontal pathology. Consequently, modulation of PMN activation might be a useful approach to periodontal therapy.

## Introduction

As in other mucosal infections, the host response to the bacteria in periodontitis is characterised by the mucosal efflux of PMNs (1–3). The PMNs influx into the crevice appears to be the first line of defence against plaque bacteria (4). The crevicular PMNs barely phagocytise (5–8), but abundantly form neutrophil extracellular traps (NETs) (4, 8). NETs are an innate immunity defence mechanism chiefly responsible for preventing the bacterial dissemination (9). They are extracellular web-like fibres generated by activated PMNs and are largely composed of nuclear constituents that disarm and kill bacteria extracellularly. NETs have a DNA backbone, but also contain many bactericidal substances, such as histones, human neutrophil elastase (NE), lysozyme, bactericidal permeability-increasing protein, human peptidoglycan-recognition protein S, and other PMN proteins (9–12). NETs bind Gram-positive as well as Gram-negative bacteria, immobilise them, and thus prevent the colonisation of new host surfaces (9). However, NETs can also be triggered by non-infectious agents (9, 13), placental microparticles (13), and inorganic implants (14) and can be harmful for the host (15–22). The capability of NETs to prevent bacterial spreading or to cause bystander damages makes it difficult to comprehend the role of NETs in periodontitis and their impact on the periodontitis pathology also remains elusive.

## Mini-Review

### Are NETs Beneficial for Periodontal Health?

Analysing the co-occurrence of periodontitis in patients with both known PMN and NETosis deficiencies may help understand the NET impact of NETs on periodontitis.

**Figure 1:**
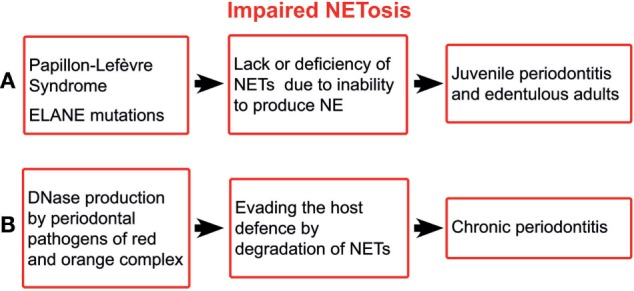
Schematic illustration of impaired NETosis in periodontitis. ELANE, the gene encoding neutrophil elastase; NETs, neutrophil extracellular traps; NE, neutrophil elastase.

Papillon–Lefèvre syndrome (PLS) is an autosomal recessive disorder characterised by palmoplantar keratosis and aggressive periodontitis. PLS results from mutations that inactivate cysteine protease cathepsin C (23), which processes various serine proteases including NE, which is an integral structural part of NETs (24, 25). Patients with PLS are either unable to form NETs or produced them in markedly reduced quantities (26, 27). Likewise, inhibitors of NE proteolytic activity, such as small β-lactam-based, cell-permeable NE inhibitors, block the NET release in neutrophils derived from healthy volunteers (25). In addition, the exogenous human secretory leucocyte protease inhibitor markedly inhibits NET formation in human neutrophils (28). The concomitance of aggressive periodontitis and the inability to form NETs suggest the indispensability of NETs for maintaining periodontal health (Figure 1A). Similarly, mutations in ELANE gene encoding NE are associated with aggressive periodontitis in the majority of patients with such mutations (29). Quite recently, the inability to form NETs has been reported for ELANE mutations (30).

Chronic granulomatous disease (CGD) is a rare primary immunodeficiency affecting the innate immune system, caused by mutations in any one of four genes encoding the subunits of the superoxide generating phagocyte NADPH oxidase, resulting in an absence or very low levels of enzyme activity (31). However, periodontitis appears to be occasional in CGD patients. Only isolated cases of periodontitis have been reported in CGD patients (32–34). A survey on 368 CGD patients reported merely nine cases of gingivitis or periodontitis (35). Individuals with inherited deficient NADPH oxidase activity, i.e., CGD patients, are capable of inducing NETosis *via* a NADPH oxidase-independent pathway; either *via* an ROS-dependent mechanism utilising ROS from other sources (36) or an ROS-independent mechanism (37). Many trigger mechanisms could be responsible for NADPH oxidase-independent NETosis in CGD patients. Thus, NADPH-oxidase-independent NETosis is stimulated by higher doses of hepoxilin A3 (38). Another possibility of CGD PMNs to produce NETs in the crevice is to utilise mitochondrial ROS (39), or other sources, e.g., ROS produced by plaque bacteria as *Streptococcus sanguinis* and *Streptococcus oralis* (40, 41). Further, *Candida albicans* (42) triggers ROS-independent NETosis as well as *Staphylococcus aureus* ROS-independent (43) and oxidant-independent NETosis (44). The fact that CGD patients are not disposed to periodontitis suggests that the oxidative burst does not appear to play a crucial role in maintaining the periodontal health, but NETs constitute the main defence. The main function of NETs appears to be that of shielding the gingiva and clearing bacteria, and their metabolic products, out of the crevice.

The ability of the major periodontal pathogens, i.e., those of red and orange complex, to produce deoxyribonucleases (45) suggests the importance of NETs for the host defence. It has been shown that extracellular nucleases enable periodontal pathogens to degrade the host NETs, leading to increased pathogenicity (46) (Figure 1B). Although the bacterial nucleases do not affect the NET proteases, the latter alone are not able to provide sufficient protection against periodontal pathogens.

The inability of patients with PLS and most of those with ELANE mutations to form NETs is concomitant with aggressive periodontitis. The ability of CGD patients to form oxidase-independent NETs is a possible explanation for the rarity of periodontitis in these patients. The most aggressive periodontal pathogens produce DNases to degrade NETs. In sum, the NET deficiency paired with aggressive periodontitis indicates the indispensability of NET for maintaining the periodontal health.

### Can NETs Be Harmful in Periodontitis?

**Figure 2:**
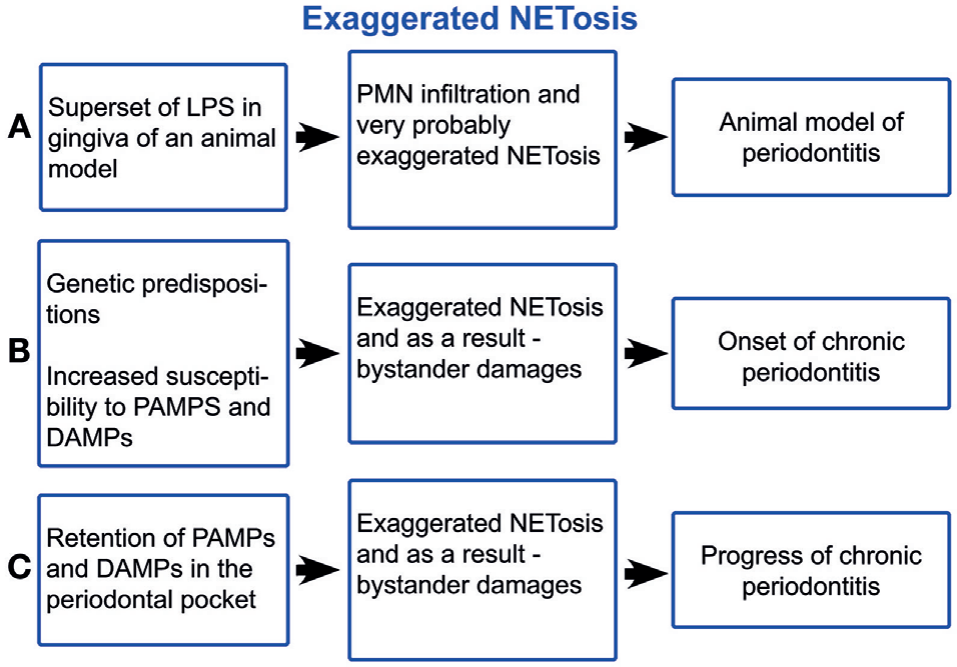
Schematic illustration of exaggerated NETosis in periodontitis. LPS, lipopolysaccharide; PMNs, polymorphonuclear neutrophil leukocytes; PAMPs, pathogen-associated molecular pattern; DAMPs, damage-associated molecular patterns.

The lipopolysaccharide (LPS) component of the cell wall of Gram-negative bacteria is an important pathogen-associated molecular pattern (PAMP) that triggers an innate immune response mainly through the activation of the toll-like receptor 4. LPS is a potent inducer of NETs (9). The supernatant of dental plaque also triggers NETosis (47). Even elevated blood plasma LPS levels have been registered in aggressive periodontitis (48) (Figure 2A). A LPS injection into the gingival tissues is a model for examining how the innate immune response to this bacterial component induces experimental periodontitis (49, 50). Histopathologically, this model is similar to other periodontitis models and to the periodontitis in humans, characterised by increased infiltration of leucocytes, higher levels of pro-inflammatory cytokines, collagen degradation, and alveolar bone resorption. Typically, a defined amount of purified bacterial LPS suspended into small micro-volumes (1–6 µl) is injected into the gingival tissues surrounding the posterior teeth of either mice or rats (51). LPS and other plaque PAMPs as well as damage-associated molecular patterns (DAMPs) activate the endothelial cells (ECs), due to the insignificant distance between high endothelial venules (HEVs) and the crevice (52, 53). Alveolar bone loss has been induced by injections of LPS from various microorganisms, including *Escherichia coli, Aggregatibacter actinomycetemcomitans*, and *Salmonella typhimurium* (51). LPS-activated ECs become leaky, as shown in the acute lung injury (54), and trigger PMN transmigration. After transmigration across the HEVs, PMNs are attracted to the crevice by PAMPs and DAMPs. LPS-stimulated PMNs selectively secrete IL8, MIP1β, and TNFα (55), which maintain EC activation. Thus, a vicious circle of PMN/HEV mutual paracrine activation may yield an exaggerated PMN response damaging the periodontal tissues. Unquestionably, the LPS effect is not restricted to HEV and PMN activations but affects the entire immunity. Thus, PMN infiltration of gingiva, PMN influx into the crevice, and subsequent NETosis is a crucial feature of periodontitis, which is an exaggerated response to the non-infectious LPS challenge. Nonetheless, PMN efflux cannot be separated from the capillaries and neither can NETosis from the PMN activation, as NETs are just a developmental stage of PMNs. The lack of resolution signals warrants the periodontal inflammation (56). The systemic effects of NETs in periodontal disease may contribute to the body’s overall inflammatory burden, worsening conditions such as diabetes mellitus, obstructive pulmonary disease, and atherosclerosis (56–60). Further, periodontitis-derived citrullinated histones (8, 61) may trigger autoimmunity, especially in rheumatoid arthritis.

### What Underlies NET Dysregulation in Periodontitis?

Genetic predispositions appear to be crucial for both the onset and the progression of periodontitis (62, 63). Chronic periodontitis occurs when untreated gingivitis progresses to the loss of the gingiva, bone, and ligament, which creates the deep periodontal “pockets” that are a hallmark of the disease (63). The pocket extends the evacuation route of the crevicular fluid, which is the blood ultra-filtrate continuously secreted in the periodontal crevice (64). NETs form a three-dimensional network entangling the particles within the crevice, notably disseminated bacteria, desquamated epithelial cells, cell debris, and fragments of biofilm matrix (4). This network is flushed out by the crevicular fluid outflow. Concomitantly with deepening the periodontal pocket, morphological changes of the pocket epithelium take place, primarily the inflammatory papillary hyperplasia. As a result, many narrow chasms between the papillae are formed, they are filled with partially and completely exfoliated epithelial cells, which cannot be efficiently flushed out by the crevicular fluid outflow (65), i.e., the pocket obstructs the evacuation of PAMPs and DAMPs out of the crevice (Figure 2B). The exaggerated NET formation causes viscosity rise (66, 67) of crevicular fluid and as a result obstruction of PAMP and DAMP evacuation. Further, NET formation is directly induced by many oral bacteria from the dental plaque (41, 47, 68, 69), neutrophil pro-inflammatory chemokines (9, 13, 70), and neutrophil-produced ROS (24). After surgery (71, 72), healing is achieved through the formation of a long junctional epithelium or a new connective tissue attachment to the previously diseased root surface, i.e., through removing the pocket obstruction of the PAMP and DAPM clearing. Thus, periodontitis occurs, given genetic susceptibility (62, 63), as consequence of the exaggerated host response to PAMP and DAPM, as the case of experimental LPS-induced periodontitis is (Figure 2C). This self-perpetuating periodontal inflammation has many common characteristics with the chronic obstructive pulmonary disease. Both diseases are characterised by heavy PMN infiltration and NETosis (73, 74), obstruction of PAMP, and DAMP evacuation and aggravation through smoking, as cigarette smoke induces NETs (75).

In cases with exaggerated production of NETs, modulation of PMN activation and NET triggering might be a helpful approach for periodontitis treatment. A broad spectrum of antioxidative substances such as flavonoids, vitamin C, 5-aminosalicylic acid, and *N*-acetyl-l-cysteine significantly inhibit the formation of ROS-dependent NETs (76). In addition, LPS effects can be reduced by gallic acid and thereby also NETosis (77). In view of the fact that some of these substances are innoxious, they might be applied topically, e.g., as dentifrice or in cases of exacerbations instilled into periodontal pockets. Indeed, further investigations are needed to estimate such possibilities.

## Conclusion

The inability of patients with PLS and most of those with ELANE mutations to form NETs indicates the role of NETs for maintaining periodontal health. The periodontal pocket formation causes clearance obstruction of PAMPs and DAMPs. The sustained PAMP and DAMP challenge triggers the exaggerated NETosis, which causes bystander damages and the disease progress. Once formed, the periodontal pocket boosts the progress of periodontitis. Modulation of exaggerated NET production by topical application of NET inhibitors might be a possible approach for prevention and treatment of periodontitis.

## Author Contributions

LV designed the study and drafted the manuscript. LV, DH, BM, and MH critically commented on the paper, contributed towards and approved the final manuscript.

## Conflict of Interest Statement

The authors declare that the research was conducted in the absence of any commercial or financial relationships that could be construed as a potential conflict of interest.

## References

[1] Attström R Egelberg J Emigration of blood neutrophils and monocytes into the gingival crevices. J Periodontal Res (1970) 5:48–55. 10.1111/j.1600-0765.1970.tb01837.x 4255146

[2] Kinane D Berglundh T Lindhe J Host–parasite interactions in periodontal disease. In: Lindhe J Karring T Lang N, editors. Clinical Periodontology and Implant Dentistry. Copenhagen: Blackwell Munksgaard (2015). 1480 p.

[3] Armitage GC Clinical evaluation of periodontal diseases. Periodontol 2000 (1995) 7:39–53. 10.1111/j.1600-0757.1995.tb00035.x 9567929

[4] Vitkov L Klappacher M Hannig M Krautgartner WD. Extracellular neutrophil traps in periodontitis. J Periodontal Res (2009) 44:664–72. 10.1111/j.1600-0765.2008.01175.x 19453857

[5] Newman HN Addison IE. Gingival crevice neutrophil function in periodontosis. J Periodontol (1982) 53:578–86. 10.1902/jop.1982.53.9.578 6752372

[6] Sigusch B Klinger G Holtz H Süss J. In vitro phagocytosis by crevicular phagocytes in various forms of periodontitis. J Periodontol (1992) 63:496–501. 10.1902/jop.1992.63.6.496 1625149

[7] Miyazaki A Kobayashi T Suzuki T Yoshie H Hara K. Loss of Fcgamma receptor and impaired phagocytosis of polymorphonuclear leukocytes in gingival crevicular fluid. J Periodontal Res (1997) 32:439–46. 10.1111/j.1600-0765.1997.tb00556.x 9266495

[8] Vitkov L Klappacher M Hannig M Krautgartner WD. Neutrophil fate in gingival crevicular fluid. Ultrastruct Pathol (2010) 34:25–30. 10.3109/01913120903419989 20070150

[9] Brinkmann V Reichard U Goosmann C Fauler B Uhlemann Y Weiss DS Neutrophil extracellular traps kill bacteria. Science (2004) 303:1532–5. 10.1126/science.1092385 15001782

[10] Cho JH Fraser IP Fukase K Kusumoto S Fujimoto Y Stahl GL Human peptidoglycan recognition protein S is an effector of neutrophil-mediated innate immunity. Blood (2005) 106:2551–8. 10.1182/blood-2005-02-0530 15956276 PMC1895263

[11] Jaillon S Peri G Delneste Y Frémaux I Doni A Moalli F The humoral pattern recognition receptor PTX3 is stored in neutrophil granules and localizes in extracellular traps. J Exp Med (2007) 204:793–804. 10.1084/jem.20061301 17389238 PMC2118544

[12] Urban CF Ermert D Schmid M Abu-Abed U Goosmann C Nacken W Neutrophil extracellular traps contain calprotectin, a cytosolic protein complex involved in host defense against *Candida albicans*. PLoS Pathog (2009) 5:e1000639. 10.1371/journal.ppat.1000639 19876394 PMC2763347

[13] Gupta AK Hasler P Holzgreve W Gebhardt S Hahn S. Induction of neutrophil extracellular DNA lattices by placental microparticles and IL-8 and their presence in preeclampsia. Hum Immunol (2005) 66:1146–54. 10.1016/j.humimm.2005.11.003 16571415

[14] Vitkov L Krautgartner W-D Obermayer A Stoiber W Hannig M Klappacher M The initial inflammatory response to bioactive implants is characterized by NETosis. PLoS One (2015) 10:e0121359. 10.1371/journal.pone.0121359 25798949 PMC4370506

[15] Hahn S Gupta AK Troeger C Rusterholz C Holzgreve W. Disturbances in placental immunology: ready for therapeutic interventions? Springer Semin Immunopathol (2006) 27:477–93. 10.1007/s00281-006-0016-5 16738957

[16] Gupta AK Hasler P Holzgreve W Hahn S. Neutrophil NETs: a novel contributor to preeclampsia-associated placental hypoxia? Semin Immunopathol (2007) 29:163–7. 10.1007/s00281-007-0073-4 17621701

[17] Kessenbrock K Krumbholz M Schönermarck U Back W Gross WL Werb Z Netting neutrophils in autoimmune small-vessel vasculitis. Nat Med (2009) 15:623–5. 10.1038/nm.1959 19448636 PMC2760083

[18] Hakkim A Fürnrohr BG Amann K Laube B Abed UA Brinkmann V Impairment of neutrophil extracellular trap degradation is associated with lupus nephritis. Proc Natl Acad Sci U S A (2010) 107:9813–8. 10.1073/pnas.0909927107 20439745 PMC2906830

[19] Caudrillier A Kessenbrock K Gilliss BM Nguyen JX Marques MB Monestier M Platelets induce neutrophil extracellular traps in transfusion-related acute lung injury. J Clin Invest (2012) 122:2661–71. 10.1172/JCI61303 22684106 PMC3386815

[20] von Bruhl M-L Stark K Steinhart A Chandraratne S Konrad I Lorenz M Monocytes, neutrophils, and platelets cooperate to initiate and propagate venous thrombosis in mice in vivo. J Exp Med (2012) 209:819–35. 10.1084/jem.20112322 22451716 PMC3328366

[21] Kahlenberg JM Carmona-Rivera C Smith CK Kaplan MJ. Neutrophil extracellular trap-associated protein activation of the NLRP3 inflammasome is enhanced in lupus macrophages. J Immunol (2013) 190:1217–26. 10.4049/jimmunol.1202388 23267025 PMC3552129

[22] Säll J Carlsson M Gidlöf O Holm A Humlén J Öhman J The antimicrobial peptide LL-37 alters human osteoblast Ca2+ handling and induces Ca2+-independent apoptosis. J Innate Immun (2013) 5:290–300. 10.1159/000346587 23406612 PMC6741588

[23] Wani AA Devkar N Patole MS Shouche YS. Description of two new cathepsin C gene mutations in patients with Papillon-Lefèvre syndrome. J Periodontol (2006) 77:233–7. 10.1902/jop.2006.050124 16460249

[24] Fuchs TA Abed U Goosmann C Hurwitz R Schulze I Wahn V Novel cell death program leads to neutrophil extracellular traps. J Cell Biol (2007) 176:231–41. 10.1083/jcb.200606027 17210947 PMC2063942

[25] Papayannopoulos V Metzler KD Hakkim A Zychlinsky A. Neutrophil elastase and myeloperoxidase regulate the formation of neutrophil extracellular traps. J Cell Biol (2010) 191:677–91. 10.1083/jcb.201006052 20974816 PMC3003309

[26] Sørensen OE Clemmensen SN Dahl SL Østergaard O Heegaard NH Glenthøj A Papillon-Lefèvre syndrome patient reveals species-dependent requirements for neutrophil defenses. J Clin Invest (2014) 124:4539–48. 10.1172/JCI76009 25244098 PMC4191054

[27] Roberts H White P Dias I McKaig S Veeramachaneni R Thakker N Characterization of neutrophil function in Papillon-Lefevre syndrome. J Leukoc Biol (2016) 100:433–44. 10.1189/jlb.5A1015-489R 26957212

[28] Zabieglo K Majewski P Majchrzak-Gorecka M Wlodarczyk A Grygier B Zegar A The inhibitory effect of secretory leukocyte protease inhibitor (SLPI) on formation of neutrophil extracellular traps. J Leukoc Biol (2015) 98:99–106. 10.1189/jlb.4AB1114-543R 25917460 PMC4467168

[29] Ye Y Carlsson G Wondimu B Fahlén A Karlsson-Sjöberg J Andersson M Mutations in the ELANE gene are associated with development of periodontitis in patients with severe congenital neutropenia. J Clin Immunol (2011) 31:936–45. 10.1007/s10875-011-9572-0 21796505 PMC3223588

[30] Thanarajasingam U Jensen MA Dorschner JM Wampler Muskardin T Ghodke-Puranik Y Purmalek M A novel ELANE mutation associated with inflammatory arthritis, defective NETosis, and recurrent parvoviral infection. Arthritis Rheumatol (2017). 10.1002/art.40314 28881492 PMC5711570

[31] Heyworth PG Cross AR Curnutte JT. Chronic granulomatous disease. Curr Opin Immunol (2003) 15:578–84. 10.1016/S0952-7915(03)00109-2 14499268

[32] Cohen MS Leong PA Simpson DM. Phagocytic cells in periodontal defense: periodontal status of patients with chronic granulomatous disease of childhood. J Periodontol (1985) 56:611–7. 10.1902/jop.1985.56.10.611 3863911

[33] Buduneli N Baylas H Aksu G Kütükçüler N. Prepubertal periodontitis associated with chronic granulomatous disease. J Clin Periodontol (2001) 28:589–93. 10.1034/j.1600-051x.2001.028006589.x 11350528

[34] Dar-Odeh NS Hayajneh WA Abu-Hammad OA Hammad HM Al-Wahadneh AM Bulos NK Orofacial findings in chronic granulomatous disease: report of twelve patients and review of the literature. BMC Res Notes (2010) 3:37. 10.1186/1756-0500-3-37 20163723 PMC2841072

[35] Winkelstein JA Marino MC Johnston RB Boyle J Curnutte J Gallin JI Chronic granulomatous disease: report on a national registry of 368 patients. Medicine (2000) 79:155–69. 10.1097/00005792-200005000-00003 10844935

[36] Nishinaka Y Arai T Adachi S Takaori-Kondo A Yamashita K. Singlet oxygen is essential for neutrophil extracellular trap formation. Biochem Biophys Res Commun (2011) 413:75–9. 10.1016/j.bbrc.2011.08.052 21871447

[37] Arai Y Nishinaka Y Arai T Morita M Mizugishi K Adachi S Uric acid induces NADPH oxidase-independent neutrophil extracellular trap formation. Biochem Biophys Res Commun (2014) 443:556–61. 10.1016/j.bbrc.2013.12.007 24326071

[38] Douda DN Grasemann H Pace-Asciak C Palaniyar NA Lipid mediator hepoxilin A3 is a natural inducer of neutrophil extracellular traps in human neutrophils. Mediators Inflamm (2015) 2015:1–7. 10.1155/2015/520871 PMC4345265 25784781

[39] Douda DN Khan MA Grasemann H Palaniyar N. SK3 channel and mitochondrial ROS mediate NADPH oxidase-independent NETosis induced by calcium influx. Proc Natl Acad Sci U S A (2015) 112:2817–22. 10.1073/pnas.1414055112 25730848 PMC4352781

[40] Okahashi N Sumitomo T Nakata M Sakurai A Kuwata H Kawabata S. Hydrogen peroxide contributes to the epithelial cell death induced by the oral mitis group of streptococci. PLoS One (2014) 9:e88136. 10.1371/journal.pone.0088136 24498253 PMC3909332

[41] Sumioka R Nakata M Okahashi N Li Y Wada S Yamaguchi M *Streptococcus sanguinis* induces neutrophil cell death by production of hydrogen peroxide. PLoS One (2017) 12:e0172223. 10.1371/journal.pone.0172223 28222125 PMC5319702

[42] Byrd AS O’Brien XM Johnson CM Lavigne LM Reichner JS. An extracellular matrix-based mechanism of rapid neutrophil extracellular trap formation in response to *Candida albicans*. J Immunol (2013) 190:4136–48. 10.4049/jimmunol.1202671 23509360 PMC3622194

[43] Björnsdottir H Dahlstrand Rudin A Klose FP Elmwall J Welin A Stylianou M Phenol-soluble modulin α peptide toxins from aggressive *Staphylococcus aureus* induce rapid formation of neutrophil extracellular traps through a reactive oxygen species-independent pathway. Front Immunol (2017) 8:257. 10.3389/fimmu.2017.00257 28337204 PMC5343011

[44] Pilsczek FH Salina D Poon KKH Fahey C Yipp BG Sibley CD A novel mechanism of rapid nuclear neutrophil extracellular trap formation in response to *Staphylococcus aureus*. J Immunol (2010) 185:7413–25. 10.4049/jimmunol.1000675 21098229

[45] Palmer LJ Chapple ILC Wright HJ Roberts A Cooper PR Extracellular deoxyribonuclease production by periodontal bacteria: deoxyribonuclease production by periodontal bacteria. J Periodontal Res (2012) 47:439–45. 10.1111/j.1600-0765.2011.01451.x 22150619

[46] Doke M Fukamachi H Morisaki H Arimoto T Kataoka H Kuwata H. Nucleases from *Prevotella intermedia* can degrade neutrophil extracellular traps. Mol Oral Microbiol (2017) 32:288–300. 10.1111/omi.12171 27476978 PMC5516193

[47] Hirschfeld J Dommisch H Skora P Horvath G Latz E Hoerauf A Neutrophil extracellular trap formation in supragingival biofilms. Int J Med Microbiol (2015) 305:453–63. 10.1016/j.ijmm.2015.04.002 25959370

[48] Shaddox LM Wiedey J Calderon NL Magnusson I Bimstein E Bidwell JA Local inflammatory markers and systemic endotoxin in aggressive periodontitis. J Dent Res (2011) 90:1140–4. 10.1177/0022034511413928 21730256 PMC3169885

[49] Abe T Hajishengallis G. Optimization of the ligature-induced periodontitis model in mice. J Immunol Methods (2013) 394:49–54. 10.1016/j.jim.2013.05.002 23672778 PMC3707981

[50] Nogueira AVB Chaves de Souza JA de Molon RS da Silva Mariano Pereira E de Aquino SG Giannobile WV HMGB1 localization during experimental periodontitis. Mediators Inflamm (2014) 2014:1–10. 10.1155/2014/816320 24692854 PMC3945472

[51] Graves DT Kang J Andriankaja O Wada K Rossa C Jr Animal models to study host-bacteria interactions involved in periodontitis. In: Kinane DF Mombelli A, editors. Frontiers of Oral Biology. Basel: KARGER (2012). p. 117–32. 10.1159/000329675 PMC3766715 22142960

[52] Zoellner H Chapple CC Hunter N. Microvasculature in gingivitis and chronic periodontitis: disruption of vascular networks with protracted inflammation. Microsc Res Tech (2002) 56:15–31. 10.1002/jemt.10009 11810703

[53] Kasprzak A Surdacka A Tomczak M Konkol M Role of high endothelial postcapillary venules and selected adhesion molecules in periodontal diseases: a review: high endothelial venules in periodontitis. J Periodontal Res (2013) 48:1–21. 10.1111/j.1600-0765.2012.01492.x 22582923

[54] Gandhirajan RK Meng S Chandramoorthy HC Mallilankaraman K Mancarella S Gao H Blockade of NOX2 and STIM1 signaling limits lipopolysaccharide-induced vascular inflammation. J Clin Invest (2013) 123(2):887–902. 10.1172/JCI65647 23348743 PMC3561818

[55] Van Dyke TE. Pro-resolving mediators in the regulation of periodontal disease. Mol Aspects Med (2017). 10.1016/j.mam.2017.04.006 28483532 PMC5660638

[56] Jeffcoat MK Hauth JC Geurs NC Reddy MS Cliver SP Hodgkins PM Periodontal disease and preterm birth: results of a pilot intervention study. J Periodontol (2003) 74:1214–8. 10.1902/jop.2003.74.8.1214 14514236

[57] Khader YS Dauod AS El-Qaderi SS Alkafajei A Batayha WQ. Periodontal status of diabetics compared with nondiabetics: a meta-analysis. J Diabetes Complications (2006) 20:59–68. 10.1016/j.jdiacomp.2005.05.006 16389170

[58] Gotsman I Lotan C Soskolne WA Rassovsky S Pugatsch T Lapidus L Periodontal destruction is associated with coronary artery disease and periodontal infection with acute coronary syndrome. J Periodontol (2007) 78:849–58. 10.1902/jop.2007.060301 17470018

[59] Kucukcoskun M Baser U Oztekin G Kiyan E Yalcin F. Initial periodontal treatment for prevention of chronic obstructive pulmonary disease exacerbations. J Periodontol (2013) 84:863–70. 10.1902/jop.2012.120399 23003917

[60] Hobbins S Chapple I Sapey E Stockley R. Is periodontitis a comorbidity of COPD or can associations be explained by shared risk factors/behaviors? Int J Chron Obstruct Pulmon Dis (2017) 12:1339–49. 10.2147/COPD.S127802 28496317 PMC5422335

[61] Janssen KMJ de Smit MJ Withaar C Brouwer E van Winkelhoff AJ Vissink A Autoantibodies against citrullinated histone H3 in rheumatoid arthritis and periodontitis patients. J Clin Periodontol (2017) 44:577–84. 10.1111/jcpe.12727 28370244

[62] Borrell LN Papapanou PN. Analytical epidemiology of periodontitis. J Clin Periodontol (2005) 32:132–58. 10.1111/j.1600-051X.2005.00799.x 16128835

[63] Kinane DF Stathopoulou PG Papapanou PN Periodontal diseases. Nat Rev Dis Primer (2017) 3:17038 10.1038/nrdp.2017.38 28805207

[64] Goodson JM Gingival crevice fluid flow. Periodontol 2000 (2003) 31:43–54. 10.1034/j.1600-0757.2003.03104.x 12656995

[65] Vitkov L Krautgartner WD Hannig M. Surface morphology of pocket epithelium. Ultrastruct Pathol (2005) 29:121–7. 10.1080/01913120590916832 16028668

[66] Picot R Das I Reid L. Pus, deoxyribonucleic acid, and sputum viscosity. Thorax (1978) 33:235–42. 10.1136/thx.33.2.235 26989 PMC470876

[67] Shak S Capon DJ Hellmiss R Marsters SA Baker CL. Recombinant human DNase I reduces the viscosity of cystic fibrosis sputum. Proc Natl Acad Sci U S A (1990) 87:9188–92. 10.1073/pnas.87.23.9188 2251263 PMC55129

[68] Palmer LJ Damgaard C Holmstrup P Nielsen CH. Influence of complement on neutrophil extracellular trap release induced by bacteria. J Periodontal Res (2016) 51:70–6. 10.1111/jre.12284 25900429

[69] Hirschfeld J Roberts HM Chapple ILC Parčina M Jepsen S Johansson A Effects of *Aggregatibacter actinomycetemcomitans* leukotoxin on neutrophil migration and extracellular trap formation. J Oral Microbiol (2016) 8:33070. 10.3402/jom.v8.33070 27834173 PMC5103672

[70] Alfaro C Teijeira A Onate C Perez G Sanmamed MF Andueza MP Tumor-produced interleukin-8 attracts human myeloid-derived suppressor cells and elicits extrusion of neutrophil extracellular traps (NETs). Clin Cancer Res (2016) 22:3924–36. 10.1158/1078-0432.CCR-15-2463 26957562

[71] Everett FG Waerhaug J Widman A Leonard Widman: surgical treatment of pyorrhea alveolaris. J Periodontol (1971) 42:571–9. 10.1902/jop.1971.42.9.571 4937198

[72] Ramfjord SP Nissle RR The modified Widman flap. J Periodontol (1974) 45:601–7. 10.1902/jop.1974.45.8.2.601 4529305

[73] Qiu Y Zhu J Bandi V Atmar RL Hattotuwa K Guntupalli KK Biopsy neutrophilia, neutrophil chemokine and receptor gene expression in severe exacerbations of chronic obstructive pulmonary disease. Am J Respir Crit Care Med (2003) 168:968–75. 10.1164/rccm.200208-794OC 12857718

[74] Obermayer A Stoiber W Krautgartner W-D Klappacher M Kofler B Steinbacher P New aspects on the structure of neutrophil extracellular traps from chronic obstructive pulmonary disease and in vitro generation. PLoS One (2014) 9:e97784. 10.1371/journal.pone.0097784 24831032 PMC4022649

[75] Qiu S-L Zhang H Tang Q Bai J He Z-Y Zhang J-Q Neutrophil extracellular traps induced by cigarette smoke activate plasmacytoid dendritic cells. Thorax (2017). 10.1136/thoraxjnl-2016-209887 28720648

[76] Kirchner T Hermann E Möller S Klinger M Solbach W Laskay T Flavonoids and 5-aminosalicylic acid inhibit the formation of neutrophil extracellular traps. Mediators Inflamm (2013) 2013:1–14. 10.1155/2013/710239 24381411 PMC3871909

[77] Haute GV Caberlon E Squizani E de Mesquita FC Pedrazza L Martha BA Gallic acid reduces the effect of LPS on apoptosis and inhibits the formation of neutrophil extracellular traps. Toxicol In Vitro (2015) 30:309–17. 10.1016/j.tiv.2015.10.005 26475966

